# ISL-1 is overexpressed in non-Hodgkin lymphoma and promotes lymphoma cell proliferation by forming a p-STAT3/p-c-Jun/ISL-1 complex

**DOI:** 10.1186/1476-4598-13-181

**Published:** 2014-07-29

**Authors:** Qiao Zhang, Zhe Yang, Zhuqing Jia, Cuiling Liu, Chen Guo, Huafei Lu, Ping Chen, Kangtao Ma, Weiping Wang, Chunyan Zhou

**Affiliations:** 1 Department of Biochemistry and Molecular Biology, School of Basic Medical Sciences, Key Laboratory of Molecular Cardiovascular Sciences, Ministry of Education of China, Peking University, 38 Xueyuan Road, 100191 Beijing, China; 2 Department of Pathology, School of Basic Medical Sciences, Peking University, 38 Xueyuan Road, 100191 Beijing, China

**Keywords:** ISL-1, Lymphomagenesis, Signal transduction, Transcriptional complex

## Abstract

**Background:**

Insulin enhancer binding protein-1 (ISL-1), a LIM-homeodomain transcription factor, is essential for the heart, motor neuron and pancreas development. Recently, ISL-1 has been found in some types of human cancers. However, how ISL-1 exerts the role in tumor development is not clear.

**Methods and results:**

The expression of ISL-1 was assessed in 211 human lymphoma samples and 23 normal lymph node samples. Immunohistochemistry results demonstrated a markedly higher expression of ISL-1 in 75% of non-Hodgkin lymphoma (NHL) samples compared with that in normal lymph nodes or Hodgkin lymphoma (HL) samples. CCK-8 analysis, cell cycle assay and xenograft model were performed to characterize the association between ISL-1 expression level and biological functions in NHL. The results showed that ISL-1 overexpression obviously promoted NHL cells proliferation, changed the cell cycle distribution *in vitro* and significantly enhanced xenografted lymphoma development *in vivo*. Real-time PCR, Western blot, luciferase assay and ChIP assay were used to explore the potential regulatory targets of ISL-1 and the results demonstrated that ISL-1 activated the c-Myc expression in NHL by direct binding to a conserved binding site on the c-Myc enhancer. Further results revealed that ISL-1 could be positively regulated by the c-Jun N-terminal kinase (JNK) and the Janus kinase/signal transducer and activator of transcription (JAK/STAT) pathways. Both the JNK and JAK/STAT signaling inhibitors could significantly suppressed the growth of NHL cells through the down-regulation of ISL-1 as demonstrated by CCK-8 and Western blot assays. Bioinformatic analysis and luciferase assay exhibited that ISL-1 was a novel target of p-STAT3 and p-c-jun. ChIP, Co-IP and ChIP-re-IP analysis revealed that ISL-1 could participate with p-STAT3 and p-c-Jun to form a p-STAT3/p-c-Jun/ISL-1 transcriptional complex that binds directly on the ISL-1 promoter, demonstrating a positive feedback regulatory mechanism for ISL-1 expression in NHL.

**Conclusions:**

Our results provide the first evidence that ISL-1 is tightly linked to NHL proliferation and development by promoting c-Myc transcription, and its aberrant expression was regulated by p-STAT3/p-c-Jun/ISL-1 complex activation.

## Introduction

Malignant lymphoma is a group of hematological malignancies, which includes Hodgkin lymphoma (HL) and non-Hodgkin lymphoma (NHL). NHL make up around 90%, and HL account for the remaining 10% of all malignant lymphomas [1]. NHL is generally classified according to its origin, that is, B-cell NHL and T/NK-cell NHL. The most common NHL subtypes by far in developed countries are diffuse large B-cell lymphoma (DLBCL) and follicular lymphoma (FL). All other NHL subtypes have a frequency of less than 10% [1]. NHL is the seventh most frequent cancer and the incidence rate has increased markedly in recent years [2]. Although some progress is being made, the fundamental abnormalities underlying NHL still remain unclear. The molecular mechanisms responsible for the etiology of NHL are poorly understood and their elucidation could improve current therapeutic approaches.

Insulin enhancer binding protein-1 (ISL-1) is a member of LIM-homeodomain family. It is previously described to play crucial roles in the development of heart, motor neuron and pancreas [3-6]. Recent studies demonstrate that ISL-1 is also involved in postnatal physiology and pathology [7-9]. More reports indicate that ISL-1 may be closely related to the occurrence of a variety of tumors. High expression level of ISL-1 is detected in a majority of pancreatic endocrine tumors [10], all four subtypes of lung cancer [11], breast cancer [12], and nearly 65% of cholangiocarcinoma [13]. Most recent study indicates that ISL-1 is a sensitive but not entirely specific marker of pancreatic neuroendocrine neoplasms (NENs) and their metastases. The overall sensitivity and specificity of ISL-1 in identifying primary pancreatic NENs is 88% and 80%, respectively [14]. Increasing evidences indicate an important role of ISL-1 in the development of some cancers. However, whether ISL-1 has any functional effect on tumorigenesis and how ISL-1 is regulated during cancer development are yet not clear.

In the present study, we investigate whether ISL-1 plays an oncogenic role in human tumors. We show that abnormal high expression of ISL-1 is significantly correlated with NHL and is specifically exhibited in 75% of human NHL samples we examined. Aberrant ISL-1 is regulated by p-STAT3/p-c-Jun/ISL-1 transcription complex and potentiates NHL cells proliferation through up-regulating c-Myc expression. Our findings reveal the feasibility of ISL-1 as a potential therapeutic target for NHL treatment.

## Results

### ISL-1 is highly expressed in 75% of human NHL samples

In our pilot study, the specimens from different types of tumors (colon, lung, breast, liver and ovarian neoplasms) were analyzed by immunohistochemical staining. The results showed a high expression level of ISL-1 in diffuse large B cell lymphoma (DLBCL, the most common lymphoma subtype and accounting for 30 ~ 40% of adult non-Hodgkin lymphoma [15]), compared with reactive lymph nodes (data not shown).

**Figure 1:**
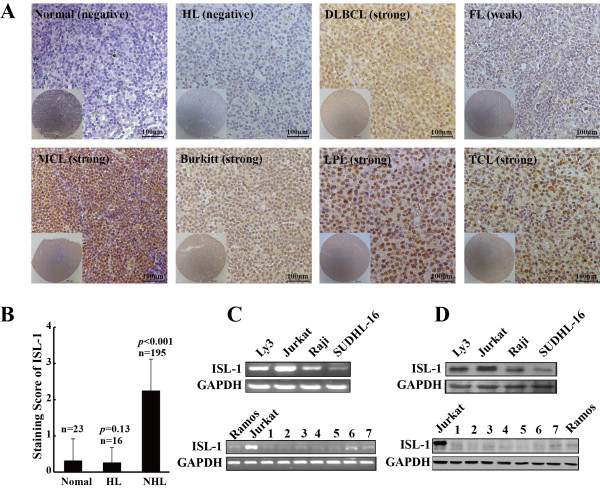
**ISL-1 is highly expressed in the majority subtypes of NHL. (A)** Immunohistochemistry for ISL-1 expression in normal lymph nodes and multiple subtypes of lymphoma specimens was performed. Representative images of ISL-1 expression level and cellular distribution in different samples are shown (200 ×). Scale bars = 100 μm. **(B)** Staining scores of ISL-1 in normal lymph nodes, HL and NHL were statistically analyzed by χ^2^ test. **(C** to **D)** The mRNA and protein levels of ISL-1 in NHL cell lines and health human peripheral white blood cells (PBC) were analyzed by RT-PCR **(C)** and Western blot **(D)** analysis. Numbers 1–7 represent PBC samples from different donors.

To examine the pathological relevance of ISL-1 in human lymphoma development, we analyzed the expression level and cellular distribution of ISL-1 in collected specimens and tissue microarrays by immunohistochemical staining. These tissue specimens included 23 normal lymph nodes and 211 lymphoma samples. The lymphoma specimens could be classified into two types: 195 NHL (159 B-cell lymphoma, 36 T-cell lymphoma) and 16 Hodgkin lymphoma (HL). As summarized in Table 1, ISL-1 expression level is markedly elevated in 75% of 195 NHL samples. Only 3 cases of normal lymph nodes exhibited moderate ISL-1 immunostaining, none of the 23 normal lymph nodes or 16 HL showed any strong positive staining for ISL-1. Figure 1A shows representative immunohistochemistry images of ISL-1 staining in human normal lymph node, HL and NHL. ISL-1 staining was predominantly detected in the nuclear of a series of NHL lymphoma cells and, to a much lesser extent, in the normal lymph nodes and HL samples. Statistical analysis revealed that there was no significant difference in the expression of ISL-1 between normal lymph nodes and HL samples (*p* = 0.13), whereas, the positive staining of ISL-1 was significantly correlated with NHLs compared with that in normal lymph nodes (*p* < 0.001) (Figure 1B).Meanwhile, we found a predominant expression of ISL-1 in a variety of NHL cell lines (Figure 1C,D). These data establish that ISL-1 expression is highly elevated in the majority of NHLs and might be tightly linked to lymphomagenesis.

**Table 1:** Immunohistochemistry of ISL-1 expression in non-Hodgkin lymphoma

**Lymphoma subtype**	**No. samples**	**Staining**			% positive*
		**Negative**	**Weak**	**Strong**	
**B-cell lymphoma (n = 159)**					
DLBCL	139	2	24	113	81
Follicular Lymphoma	8	1	5	2	25
Mantle cell lymphoma	4	0	1	3	75
Burkitt lymphoma	3	0	1	2	67
Lymphoplasmacytic lymphoma	5	0	1	4	80
**T-cell lymphoma**	36	3	10	23	64
**Total**	195	6	42	147	75

*Positive percentage is determined by number of strong staining samples over the total number of samples.

### ISL-1 promotes proliferation of NHL cells *in vitro* and enhances xenografted lymphoma development *in vivo*

**Figure 2:**
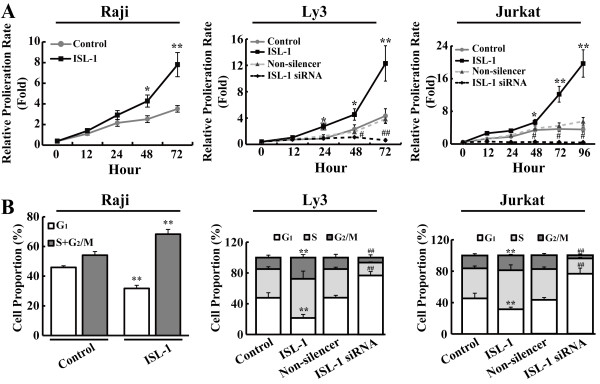
**ISL-1 promotes NHL cells proliferation and affects cell-cycle phase distributions. (A)** The relative proliferation rate of stably transfected cells was determined by CCK-8 assay at indicated time post-seeding. **(B)** Cell cycle distributions were analyzed by flow cytometry. The data represent three independent experiments. Each bar represents mean ± SD. *p* values were calculated using a Student *t*-test (*p < 0.05, **p < 0.01, ^#^*p* < 0.05, ^##^*p* < 0.01 vs. each control). (Control, ISL-1, Non-silencer and ISL-1 siRNA represent the cells transfected with pcDNA3.1, pcDNA3.1-ISL-1, pLL3.7-Non-silencer or pLL3.7-ISL1-siRNA plasmid, respectively)

We have previously shown that ISL-1 promoted the proliferation of adult pancreatic islets cells [6]. We wonder whether up-regulated ISL-1 in NHL plays a role in promoting NHL cells proliferation and tumorigenesis. Therefore, Raji, Jurkat and Ly3 were electroporated with pcDNA3.1-ISL1, or pLL3.7-ISL1-siRNA plasmid to establish stable ISL-1 overexpressing or knockdown NHL cell lines. ISL-1 expression level in stably transfected NHL cell lines was measured by Western blot analysis and the degree of ISL-1 expression changes was analyzed by gray scanning using Bio-Rad Quantity One software on the images from 3 independent experiments. The results showed that the expression level of ISL-1 was ameliorated approximately 7 folds in ISL-1 overexpressed Raji cells (Additional file 1: Figure S1A) and around 2.7 folds in ISL-1 overexpressed Ly3 and Jurkat cells (Additional file 1: Figure S1B,C), while the level of ISL-1 was attenuated to less than 10% in ISL-1 knockdown Ly3 and Jurkat cells (Additional file 1: Figure S1B,C), indicating that both overexpression and knockdown cell lines are successfully established. When ISL-1 protein level was up or down-regulated, notable promotion or inhibition of cell growth were observed in corresponding cell lines (Figure 2A). To further determine the role of ISL-1 on proliferation of NHL cells, the cell cycle profiles were analyzed. Compared with the control, Raji, Ly3 and Jurkat cells with ISL-1-overexpression showed a decreased cell population in G_1_ phase and a remarkably increased cell population in the S and G_2_/M phases. Conversely, Ly3 and Jurkat cells with ISL-1 knockdown exhibited an increase in the proportion of cells in G_1_ phase and a decrease in the proportion of cells in S and G_2_/M phases (Figure 2B). These results indicate that ISL-1 could significantly change the cell cycle dynamics and thus promote NHL cells proliferation.

**Figure 3:**
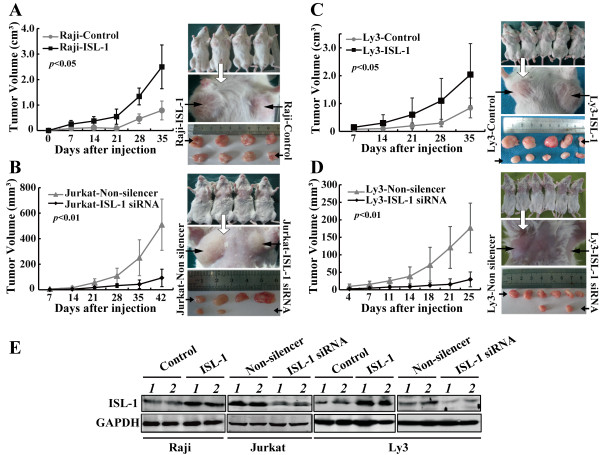
**ISL-1 enhances xenografted lymphoma development *in vivo*. (A** to **D)** NOD-SCID (nonobese diabetic/severe combined immunodeficient) mice were injected s.c. with different NHL cells that were stably transfected with pcDNA3.1 (Control), or pcDNA3.1-ISL-1 (ISL-1) construct **(A,C)**, pLL3.7-Non-silencer or pLL3.7-ISL1-siRNA plasmid **(B,D)**. The tumor size was monitored at indicated days post-injection. Statistical analysis was carried out with 2-way ANOVA. **(E)** The mice were killed after the last measurement of tumor volume, whole-cell lysate of 2 tumor samples of each group were prepared and subjected to Western blot analysis for ISL-1 level detection. GAPDH served as an internal control.

To further confirm whether ISL-1 could promote tumor growth *in vivo*, we used the SCID mice xenograft model to study the impact of ISL-1 on NHL genesis and development. We found that the initiation and the growth of tumor were significantly earlier and faster with ISL-1 overexpressing cells than those with the control cells (Figure 3A,C). Conversely, the tumor growth was obviously impaired with ISL-1 knockdown cells (Figure 3B,D). After the last measurement, the tumors were isolated and weighed. The ISL-1-overexpressing cells produced significantly larger and heavier tumors than the control cells, in contrast, the ISL-1-knockdown cells produced smaller and lighter tumors compared with the control cells (Additional file 2: Figure S2). We further compared the expression of ISL-1 in the tumor tissues isolated from the mice. As shown in Figure 3E, the protein level of ISL-1 in the tumors was positively correlated with the tumor volumes in each group. Therefore, our animal experiments confirm that ISL-1 potentiates NHL growth *in vivo*.

Collectively, *in vitro* and *in vivo* results indicate that overexpression of ISL-1 promotes NHL cells proliferation and enhances lymphoma development, whereas knockdown of ISL-1 attenuates NHL cells proliferation and inhibits xenograft growth.

### ISL-1 stimulates NHL cell proliferation through the up-regulation of c-Myc expression

**Figure 4:**
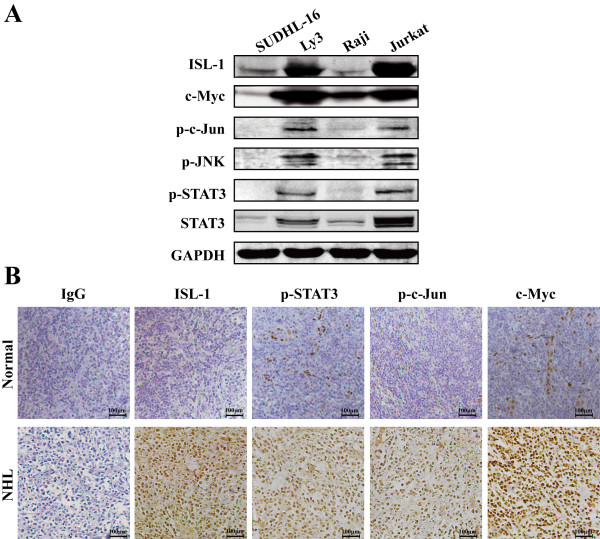
**The expression of ISL-1 is positively correlated to the expression of p-STAT3, p-c-Jun and c-Myc. (A)** NHL cell lines were analyzed by Western blot with indicated antibodies. **(B)** Immunohistochemistry for ISL-1, p-STAT3, p-c-Jun and c-Myc expression were performed in multiple specimens of normal lymph nodes (top panel) and NHL patients (bottom panel). Representative images of ISL-1, p-STAT3, p-c-Jun and c-Myc expression levels and cellular distributions in different samples are shown (200 ×). Scale bars = 100 μm

**Figure 5:**
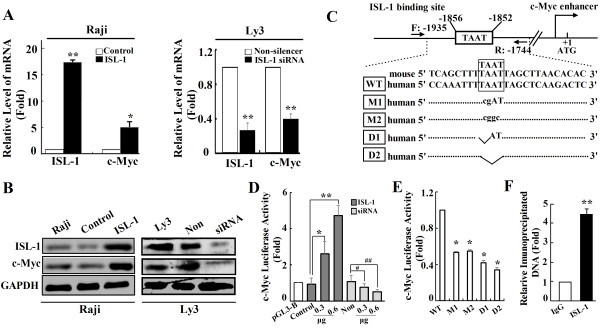
**ISL-1 promotes the expression of c-Myc in NHL cell lines. (A** to **B)** The expression of ISL-1 and c-Myc were analyzed at both mRNA and protein levels by real-time RT-PCR **(A)** and Western blot **(B)** in Raji cells with stable ISL-1 overexpression and Ly3 cells with stable ISL-1 knockdown. **(C)** Consensus binding site (TAAT box) for ISL-1 on the human c-Myc enhancer was analyzed by MatInspector software. The mutant sequences are presented and they were used to construct mutant *c-Myc-luc*. **(D** to **E)** The transcriptional activity of ISL-1 on *c-Myc-luc* wide type **(D)**, mutants or deletions **(E)** was analyzed by luciferase reporter assay in HeLa cells. (“WT”, “M” and “D” represent the plasmid of *c-Myc-luc* wide type, mutant, or deletion, respectively.). Non, WT and ctrl served as the control in corresponding experiments. **(F)** ISL-1 recruited on c-Myc promoter was analyzed by ChIP assay. Soluble chromatin was prepared from Ly3 cells followed by immunoprecipitation with the antibody against ISL-1 and the normal IgG served as a control. The DNA extractions were amplified using the primers that covered the ISL-1 binding sites on c-Myc enhancer region by real-time PCR. The data represent 3 independent experiments, each performed in triplicate. Each bar represents mean ± SD. *p* values were calculated using a Student *t*-test (*p < 0.05, **p < 0.01, ^#^*p* < 0.05 vs. the control).

To explore the mechanism of ISL-1-stimulated NHL cell proliferation, bioinformatic analysis was performed with professional MatInspector software and refFlat Database to identify the downstream target genes of ISL-1. Several putative genes, including CyclinD1, BCL-6 and c-Myc were identified for further investigation, as these genes contain conserved ISL-1 binding sequences (YTAATGR) on the upstream of the ATG translation start site [16-18]. More importantly, they are remarkably related to the pathogenesis of NHL as previously reported [16-18]. However, the expression of CyclinD1 and BCL-6 did not show a predicted correlation with ISL-1 in NHL cells (data not shown). Therefore, we focused on c-Myc in the rest investigations.Western blot results showed that the basal expression level of c-Myc was positively correlated with the expression level of ISL-1 in NHL cell lines (will be discussed later in Figure 4A). Moreover, further results indicated that the overexpression of ISL-1 increased the expression of c-Myc at both mRNA and protein levels in Raji cells (Figure 5A, B left panel). Whereas, the significant decrease of c-Myc expression was associated with the knockdown of ISL-1 as compared with those in the control Ly3 cells (Figure 5A,B right panel). These results show that ISL-1 could act as a transcriptional activator of c-Myc.

Furthermore, we uncovered that c-Myc is a direct transcriptional target of ISL-1. Bioinformatic analysis revealed a conserved ISL-1 binding site (TAAT) at -1856 ~ -1852 bp upstream of the ATG translation start site on the c-Myc enhancer region (Figure 5C). Luciferase assay with *c-Myc-luc* (a c-Myc luciferase reporter construct that contains the binding site for ISL-1 on the c-Myc enhancer) showed the stimulated *c-Myc-luc* activity in ISL-1-overexpressing cells in a dose-dependent manner, whereas a significant decrease of *c-Myc-luc* activity was seen in ISL-1-knockdown cells (Figure 5D). The constructs containing the mutant or deleted ISL-1 binding site on the c-Myc enhancer (Figure 5C), *c-Myc-luc* M1 (“TAAT” mutated to “cgAT”), *c-Myc-luc* M2 (“TAAT” mutated to “cggc”), *c-Myc-luc* D1 (“TAAT” with “TA” deleted) and *c-Myc-luc* D2 (“TAAT” completely deleted), exhibited a significant decrease of luciferase activity compared to the wild type *c-Myc-luc* (Figure 5E).

To determine if ISL-1 could occupy the c-Myc enhancer region *in vivo*, a specific primer covering the potential ISL-1 binding site located between -1935 and -1744 bp of c-Myc enhancer were designed (Figure 5C) and used for chromatin immunoprecipitation (ChIP) assay in Ly3 cells. As shown in Figure 5F, ISL-1 was recruited to the c-Myc enhancer about four folds as compared with IgG, suggesting that ISL-1 could bind on the c-Myc enhancer *in vivo*. These results indicate that ISL-1 is a direct regulator of c-Myc transcription in NHL cells.

Taken together, ISL-1 promotes NHL cells proliferation possibly via the activation of the c-Myc enhancer and thus increasing its expression.

### p-c-Jun and p-STAT3 contribute to the up-regulation of ISL-1 expression in NHL cells

To explore the molecular regulatory mechanism for ISL-1 up-regulation, bioinformatic analysis was used to identify the potential regulatory factors that could bind on the transcriptional regulatory region of ISL-1. Relevant conserved binding sites of symbolic transcriptional factors, specifically pointing to major pathways such as WNT, MAPK/ERK, p38 MAPK, SAPK/JNK and JAK/STAT, were identified on the ISL-1 transcriptional regulatory region. Previous reports show that ISL-1 is regulated by or interacts with these signal pathways in different physiological or pathological processes and all these signal pathways could contribute to malignant progression of NHLs [19-25]. However, whether the up-regulated expression of ISL-1 in NHLs is mediated by these signal pathways needs to be elucidated.

To explore which signal pathway is involved in ISL-1 up-regulation in NHL, Western blot was used to analyze the impact of inhibitors or activators of the above signaling pathways on ISl-1 expression. The results showed that both JNK signaling inhibitor SP600125 and JAK/STAT signaling inhibitor STATTIC could notably reduce the expression of ISL-1 at protein level. Other inhibitors or activators exhibited little effect on the expression level of ISL-1 (Additional file 3: Figure S3). Therefore, we suppose that the expression of ISL-1 may be modulated by JNK and JAK/STAT signal pathways.

**Figure 6:**
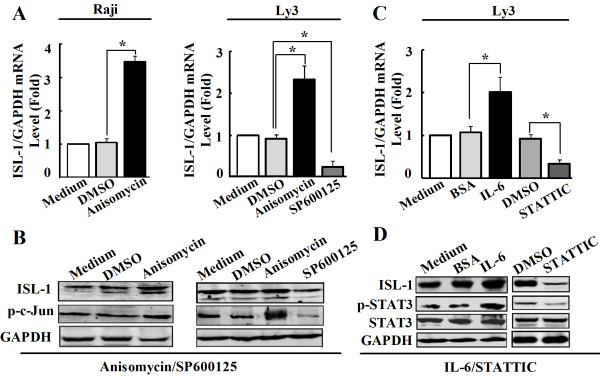
**p-c-Jun and p-STAT3 contribute to ISL-1 overexpression in NHL cells. (A** to **B)** real-time RT-PCR **(A)** and Western blot **(B)** show the expression changes of ISL-1 in Raji and Ly3 cells after treated with JNK signaling pathway activator (Anisomycin, 15 ng/ml) or inhibitor (SP60012, 10 μM) for 6 h. **(C to D)** real-time RT-PCR **(C)** and Western blot **(D)** show the expression changes of ISL-1 in Ly3 cells after treated with JAK/STAT signaling pathway activator (IL-6, 4 ng/ml) or inhibitor (STATTIC, 6 μM) for 24 h. Each bar represents mean ± SD from three samples. *p* values were calculated using a Student *t*-test (*p < 0.05, vs. the control).

As we known, p-c-Jun and p-STAT3 belong to JNK and JAK/STAT signal pathways, respectively. They are the most important functional activators for the signaling transduction and closely link to lymphoma cell survival, proliferation and transformation [21,24,26-29]. To verify how ISL-1 is regulated by JNK and JAK/STAT signal pathways, we first analyzed the basal expression levels and correlations of p-c-Jun, p-STAT3, along with ISL-1 and the prominent oncogenic protein c-Myc in a series of NHL cell lines and numbers of human NHL tissue specimens. The results of Western blot showed that the p-c-Jun and p-STAT3 were readily detectable and positive consistent with the expression level of ISL-1 and c-Myc in all these cell lines (Figure 4A). We then analyzed the expression of ISL-1, p-c-Jun, p-STAT3 and c-Myc at protein level on 35 cases of human NHL and 10 cases of human normal lymph node by immunohistochemical staining. As shown in Figure 4B, p-c-Jun, p-STAT3 and c-Myc staining were considerably stronger in NHL than in normal lymph node, in parallel with the pattern observed for ISL-1 in NHL. Pearson correlation analysis revealed that the expression level of ISL-1 protein is strongly correlated with p-STAT3, p-c-Jun and c-Myc protein levels in human NHL samples surveyed (Pearson correlation coefficient r = 0.737, 0.501, 0.803 respectively, all *p* < 0.001). These data indicate that increased coexpression of ISL-1, p-STAT3, p-c-Jun and c-Myc may be associated with the development of NHL. The above results indicate that JNK and JAK/STAT signaling pathways are likely to promote ISL-1 expression through the constitutively activated p-c-Jun and p-STAT3.Further analysis showed that the significantly increased ISL-1 expression was positively associated with the activation of p-c-Jun or p-STAT3, after treated with JNK or JAK/STAT activator (Anisomycin or IL-6). Conversely, after treated with JNK or JAK/STAT inhibitor (SP600125 or STATTIC), the expression of ISL-1 was obviously decreased (Figure 6). These results show that persistent activation of p-c-Jun and p-STAT3 lead to the aberrant transcription of ISL-1 in NHL cells.

### Inhibition of JNK and JAK/STAT pathways suppresses NHL cells proliferation via down-regulating ISL-1 expression

**Figure 7:**
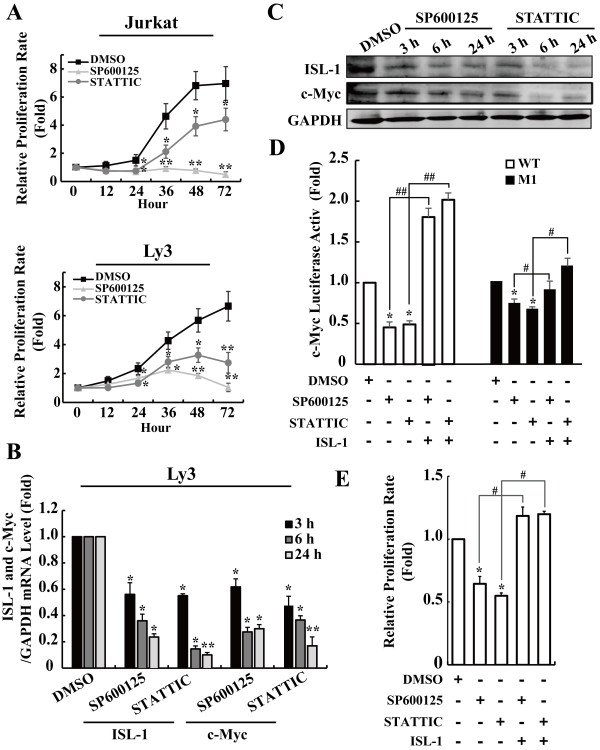
**JNK or JAK/STAT signaling inhibitors inhibit NHL cells proliferation through down-regulating ISL-1 expression. (A)** The relative proliferation rate of lymphoma cell lines were measured using CCK-8 analysis after treated with JNK signaling pathway inhibitor (SP60012, 10 μM) or JAK/STAT signaling pathway inhibitor (STATTIC, 6 μM). The cell treated with DMSO were used as the control. **(B** to **C)** The effect of SP600125 (10 μM) and STATTIC (6 μM) on ISL-1 and c-Myc expression at both mRNA **(B)** and protein levels **(C)** were analyzed by real-time RT-PCR and Western blot. The cells treated with DMSO at different time point were used as the corresponding control. **(D)** The luciferase activity of *c-Myc-luc* (wide type or M1) was measured in Ly3 cells with or without ISL-1 transcfection after treated with 10 μM SP600125 or 6 μM STATTIC for 24 h. **(E)** The growth inhibition of Ly3 cells with or without ISL-1 transcfection was measured by CCK-8 analysis after treated with 10 μM SP600125 or 6 μM STATTIC for 24 h. The data represent three independent experiments. Each bar represents mean ± SD. *p* values were calculated using a Student *t*-test (*p < 0.05, **p < 0.01, ^#^*p* < 0.05, ^##^*p* < 0.01 vs. each control).

We have shown that both JNK and JAK/STAT signaling inhibitors can suppress ISL-1 expression. However, it is not clear whether these inhibitors could affect the proliferation of NHL cells by decreasing ISL-1 expression level. CCK-8 analysis was firstly performed to analyze the influence of JNK and JAK/STAT inhibitors on NHL cells proliferation. As shown in Figure 7A, both SP600125 and STATTIC could significantly decrease the proliferation rate of NHL cells.

To further investigate whether ISL-1 was involved in the inhibition of tumor cells proliferation mediated by these inhibitors, Ly3 cells were treated with SP600125 or STATTIC to inhibit the phosphorylation of c-Jun or STAT3. As shown in Figure 7B, C and (Additional file 4: Figure S4), a distinct decreased expression of ISL-1 was correlated to SP600125 or STATTIC-induced inhibition of p-c-Jun or p-STAT3. Additionally, the expression change of c-Myc was also consistent with the change pattern of ISL-1. These data indicate that the JNK and JAK/STAT pathways regulate c-Myc expression and NHL cells proliferation, which may be partly through the regulation of ISL-1 expression.

To explore this further, luciferase assay was used to analyze the impact of SP600125 or STATTIC on the luciferase activity of *c-Myc-luc*. The inhibition of JNK and JAK/STAT pathways significantly decreased *c-Myc-luc* (wild type) activity, and the overexpression of ISL-1 could recover the effect mediated by the inhibitors of JNK and JAK/STAT pathways (Figure 7D left panel). Whereas, the mutant constructs *c-Myc-luc* M1 exhibited much smaller extent of luciferase activity changes (Figure 7D right panel). These results support that ISL-1 is involved in the JNK and JAK/STAT regulation on c-Myc expression. We next assessed whether the expression level of ISL-1 could influence the effects of p-c-Jun and p-STAT3 on the proliferation rate of Ly3 cells. As shown in Figure 7E, both p-c-Jun and p-STAT3 inhibitors could significantly suppress the proliferation of Ly3 cells transfected with the control vectors, while, the growth inhibition mediated by p-c-Jun and p-STAT3 inhibitors could be rescued in ISL-1-overexpressing cells, which further demonstrates the effect of ISL-1 on the proliferation of cells.

Collectively, JNK and JAK/STAT signaling inhibitors suppress NHL cells proliferation possibly through down-regulating ISL-1 expression. Therefore, ISL-1 may serve as a new target molecule for NHL treatment.

### p-STAT3/p-c-Jun/ISL-1 forms a transcriptional complex and binds directly to the ISL-1 promoter in NHL cells

**Figure 8:**
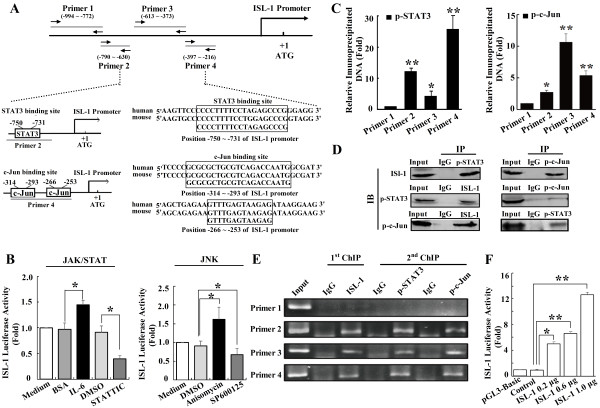
**p-STAT3/p-c-Jun/ ISL-1 forms a transcriptional complex and binds directly to ISL-1 promoter. (A)** Consensus binding sites for p-STAT3 and p-c-Jun on the ISL-1 promoter were analyzed by Matinspector software. **(B)** The luciferase activity of *ISL-1-luc* was analyzed by luciferase reporter assay in Ly3 cells after treated with IL-6 (4 ng/ml), STATTIC (6 μM), Anisomycin (15 ng/ml) or SP600125 (10 μM) for 24 h. **(C)** ChIP assay was performed with anti-p-STAT3 Ab (left panel) or anti-p-c-Jun Ab (right panel) for immunoprecipitation using chromatin harvested from Ly3 cells. The DNA extractions were amplified using the primers that cover the p-STAT3 (primers 2) or p-c-Jun (primers 4) binding sites, or control primers (primers 1, 3) on the ISL-1 promoter by real-time PCR with normal IgG as a control. **(D)** Co-IP assay was performed in Ly3 for the transcriptional complex recruited on the ISL-1 promoter. **(E)** ChIP-re-IP assay was performed first with anti-ISL-1 Ab or rabbit IgG Ab and then with anti-p-STAT3, anti-p-c-Jun or IgG Abs for immunoprecipitation using chromatin harvested from Ly3 cells. **(F)** The transcriptional activity of ISL-1 on *ISL-1-luc* was analyzed in Ly3 cells by luciferase reporter assay. The data represent three independent experiments. Each bar represents mean ± SD. *p* values were calculated using a Student *t*-test (*p < 0.05, **p < 0.01 vs. the control).

The above results have shown that p-STAT3 and p-c-Jun could increase the expression level of ISL-1 to promote the proliferation of NHL cells. However, it is unknown how p-STAT3 and p-c-Jun control ISL-1 expression. Bioinformatic analysis showed that the core transcriptional regulatory region of ISL-1 (-1000 ~ ATG) [30] contains conserved p-STAT3 and p-c-Jun binding sites (Figure 8A). Luciferase assay with *ISL-1-luc*, a ISL-1 luciferase reporter construct containing the binding site of c-Jun and STAT3, was performed in Ly3 cells treated with IL-6/STATTIC (JAK/STAT signaling activator/inhibitor) or Anisomycin/SP600125 (JNK signaling activator/inhibitor), respectively. As shown in Figure 8B, *ISL-1-luc* activity was increased in Anisomycin or IL-6 treated cells. Whereas a significant decrease of *ISL-1-luc* activity could be observed after SP600125 or STATTIC treatment. These results further demonstrate that both JNK and JAK/STAT signaling pathways are able to activate the ISL-1 transcription effectively.To confirm whether p-STAT3 and p-c-Jun bind to the ISL-1 regulatory region, a set of primers covering the ISL-1 promoter region between -994 and -216 were designed for real-time PCR in ChIP assay (Figure 8A). The ChIP analysis showed that p-STAT3 was recruited to the region of ISL-1 promoter covered by primer 2 (-790 ~ -630) by approximately 12 folds (Figure 8C left panel), and p-c-Jun was recruited to the region of ISL-1 promoter covered by primer 4 (-397 ~ -216) by about 6 folds (Figure 8C right panel), respectively, as compared with primer 1 (-994 ~ -772) as the control. Interestingly, we also observed magnificent enrichment of p-STAT3 at the p-c-Jun binding region (primer 4 covered region, Figure 8C left panel), p-c-Jun at the p-STAT3 binding region (primer 2 covered region, Figure 8C right panel), and both p-STAT3 and p-c-Jun at the primer 3 (-613 ~ -373) covered region (Figure 8C left and right panel). Therefore, we suppose that p-STAT3 possibly cooperate with p-c-Jun and synergistically regulate ISL-1 expression in NHL cells.

According to previous reports, p-STAT3 could interact with p-c-Jun to regulate MMP-1 [31], MMP-7 [32] or other genes expression in human cancers [33]. Meanwhile, the cooperation and co-localizations between p-STAT3 and ISL-1 [25,34], p-c-Jun and ISL-1 [23] are also authenticated in different genes transcription. These evidences promote us to hypothesize that p-STAT3, p-c-Jun and ISL-1 may form a transcriptional activation complex that regulates the expression of ISL-1 by direct binding to the ISL-1 promoter.To verify this hypothesis, co-immunoprecipitation (Co-IP) and ChIP-re-IP were performed to analyze whether p-STAT3, p-c-Jun and ISL-1 could form a complex and bind directly on the ISL-1 promoter. Co-IP results demonstrate that one component of the presumptive complex could co-immunoprecipitate with all of the other components, supporting the existence of this complex (Figure 8D). Furthermore, ChIP-re-IP analysis confirmed that p-STAT3, p-c-Jun and ISL-1 indeed existed in the same protein complex and co-localized on the primer 2 and primer 4 covered region of ISL-1 promoter (Figure 8E). These results reveal that p-STAT3, p-c-Jun and ISL-1 could form a transcriptional activation complex on the ISL-1 promoter, which further indicates that there might be a positive feedback loop to contribute to ISL-1 up-regulated expression in NHL cells.

To determine whether ISL-1 is involved in the positive feedback loop on the ISL-1 transcription, luciferase assay was performed with *ISL-1-luc*. As shown in Figure 8F, *ISL-1-luc* activity was increased in a dose-dependent manner in ISL-1-overexpressing Ly3 cells, indicating, for the first time, that ISL-1 could promote its own expression in NHL cells and therefore to form a positive feedback.

Collectively, these results indicate that ISL-1 may have a positive feedback regulation: p-STAT3 and p-c-Jun up-regulate ISL-1 expression, then ISL-1 form a complex with p-STAT3 and p-c-Jun to participate ISL-1 overexpression. The consequence is to promote the proliferation of NHL cells.

## Discussion

NHL is the most common lymphoid malignancy in adults, accounting for ~70,000 new cases each year in the US [2]. The aggressive NHL, which is, pathophysiologically and clinically, a very heterogeneous disease, differs significantly in its dependence on signaling pathways, and responses differently to current standard therapies [35]. Therefore, a common molecular target functionally associated with non-Hodgkin lymphomagenesis is required to be identified to develop effective therapeutic approaches that will improve the clinical outcome of patients with different subtypes of NHL. In this study, we found that an elevated expression of ISL-1 in 75% of NHL samples examined, and further studies provided evidences that aberrant expression of ISL-1 significantly correlated with NHLs and might be a potential therapeutic target for NHL treatment. However, the classification of NHL is very complicated. According to the World Health Organization (WHO) Classification, NHL could be classified into 36 subtypes (21 of B cell and 15 of T-cell type), excluding entities of uncertain malignant potential [1]. The most dominant forms of NHL are diffuse large B-cell lymphoma (about 30%) and follicular lymphoma (about 20%). All other NHL subtypes have a frequency of less than 10% [1]. In parallel with the proportion of each NHL subtype, we performed immunohistochemical analyses for ISL-1 in 195 primary lymphoma tissue specimens, including 159 B-cell lymphoma and 36 T-cell lymphoma samples. As summarized in Table 1, ISL-1 was remarkably over-expressed in 81% of 139 DLBCL samples. Meanwhile, although strong positive staining for ISL-1 was identified in 25% of 8 follicular lymphoma (FL) and 67% of 3 Burkitt lymphoma samples, respectively, the total numbers of those specimens examined were small and awaited larger confirmatory studies. In the further study, we applied the commonly used Raji (Burkitt cell line), Jurkat (T-cell leukemia/lymphoma cell line) and Ly3 (DLBCL cell line) in multiple experiments to represent NHLs [24,36,37]. However, it should not be ignored that the molecular pathogenesis of each subtype are not completely identical, which may be the possible reasons for the 25% of ISL-1 negative NHLs. Moreover, it has been reported that in addition to *de novo* DLBCL, 30-40% of FL, a low-grade NHL, will transform to an aggressive DLBCL [38,39]. According to the immunohistochemical analyses, we were able to show a significantly elevated level of ISL-1 in the vast majority (81%) of DLBCL. In contrast, we did not observe significant changes of ISL-1 in most of the indolent lymphoid malignancies examined, such as FL (25%), indicating that ISL-1 expression level might be correlated with the progression and degree of malignant NHLs.

We previously showed that ISL-1 could stimulate pancreatic islet cells growth and prevent adult pancreatic islet cells from reactive oxygen species (ROS) induced apoptosis [6]. In this study, we found that abnormally up-regulated expression of ISL-1 in NHL cells obviously facilitated cell proliferation *in vitro* and significantly enhanced lymphoma development *in vivo*, providing the first evidence that ISL-1 could promote non-Hodgkin lymphomagenesis and specific disruption of ISL-1 could be capable of inhibiting NHL cells with high endogenous levels of ISL-1. Our previous report reveals that c-Myc and CyclinD1 are novel downstream targets of ISL-1 and are involved in ISL-1 regulation on the proliferation of adult islet cells [6]. However, in NHL cells, ISL-1 could regulate c-Myc but had minimal effect on CyclinD1. These implied that c-Myc must be a more potent downstream factor of ISL-1 to mediate proliferation effects in lymphoma tumorigenesis.

The proto-oncogene c-Myc has been linked to a diverse range of cellular functions, such as cell cycle regulation, proliferation, differentiation and metabolism. Not surprisingly, aberrant c-Myc signaling has been observed to promote cell transformation and tumor progression in human cancers [40]. According to previous reports, c-Myc overexpression has not only been described as a defining feature and the driving oncogene for Burkitt lymphoma, but also been recognized in mantle cell lymphoma (MCL), DLBCL and other NHLs [18,41-43]. c-Myc overexpression in human tumors has been attributed to transcriptional regulation, gene amplification, as well as genomic translocation [40]. However, in most NHLs, the reason for c-Myc up-regulated expression has not been clearly elucidated. In this study, we show that ISL-1 is recruited to the transcriptional region of the c-Myc gene and activate its expression, which shed light on the mechanism underlying the c-Myc dysregulation and clinical lymphomagenesis.

The c-Jun N-terminal kinase (JNK) [21,26,28] and Janus kinase/signal transducer and activator of transcription (JAK/STAT) [29,44-47] signaling pathways, which are predicted to modulate ISL-1 expression, have been reported to link to the oncogenic process of a variety of lymphoma subtypes, making them appealing targets for pathway-directed cancer therapy. The application of specific signaling pathways’ activators and inhibitors demonstrated the correlation between JNK pathway and ISL-1, as well JAK/STAT pathway and ISL-1 expression [21,24]. Figure 6 showed that ISL-1 expression was increased by elevated c-Jun and STAT3 phosphorylation in Raji and Ly3 cells, respectively. Reciprocally, attenuated p-c-Jun and p-STAT3 in these cells resulted in a decreased expression of ISL-1. Furthermore, Pearson correlation analysis also revealed strong correlation between the expression level of ISL-1 with p-STAT3 and p-c-Jun protein level in human NHL samples. These data unequivocally linked ISL-1 expression level with JNK and JAK/STAT signaling pathways.

Many reports suggest that c-Myc is a downstream effector of JNK or STAT3 signaling and c-Myc protein level in NHL cells could be reduced in the presence of JNK-specific siRNA or STAT3 shRNA [21,24]. However, it remains to be determined whether p-c-Jun and p-STAT3 regulate the c-Myc expression directly or indirectly. Interestingly, our data suggested that JNK and JAK/STAT pathways could corporately regulate c-Myc expression and promote lymphoma growth through up-regulating the level of ISL-1. The overexpression of ISL-1 could rescue the growth suppression effect mediated by JNK and JAK/STAT inhibitors, indicating that ISL-1 is necessary for p-c-Jun and p-STAT3 effects. Therefore, ISL-1 might be an important common mediator of c-Myc and JNK-JAK/STAT signaling pathways in the progression of NHL. In terms of regulatory mechanism of NHL, we demonstrated that ISL-1 expression was regulated by both JNK and JAK/STAT signaling pathways, p-STAT3/p-c-Jun/ISL-1 could form a transcriptional complex and bind directly to the ISL-1 promoter, indicating that ISL-1 might has a positive feedback regulation. These conclusions are consistent with previous reports that striking coincidences for concerted aberrant activation of both STAT3 and c-Jun in human cancer specimens are observed [31], and c-Jun or c-Myc is required for the transforming activity of STAT3 in tumorgenesis [32,48]. Taken together, our results reveal a functional linkage between JNK and JAK/STAT signaling and the oncogenic roles of ISL-1 and c-Myc in NHL.

## Conclusions

Overall, in this study, we extend the knowledge about the crucial roles of ISL-1. Our findings document that ISL-1 is highly expressed in NHL and plays an oncogenic role in lymphomagenesis. Aberrant ISL-1 could stimulate cell proliferation and xenograft growth by activating c-Myc transcription. Moreover, JNK and JAK/STAT pathways contribute to ISL-1 dysregulation. Our study identifies a specific and novel function of ISL-1 in NHL development and suggests that the ISL-1 suppression represents a potential target for NHL treatment.

## Materials and methods

### Immunohistochemistry and immunohistologic analysis

All human samples were obtained from the Department of Pathology, School of Basic Medical Sciences, Peking University with the informed consent and with the approval from the Research Ethics Committee of Peking University (IRB 00001052–13014). The lymphoma tissue microarrays (TMA) 203a and LY2086a were bought from US Biomax (Rockville, MD, USA). Collected specimens and TMA were subjected to immunohistochemistry (IHC) analysis using the Enovision Detection Kit/DAB (GK500705, DAKO A/S, Glostrup, Denmark) according to the manufacturer’s protocol with the indicated antibody: mouse monoclonal anti-ISL-1 (ab86472, Abcam, Hong Kong, China); rabbit polyclonal anti-c-Myc and anti-phospho-c-Jun (Ser63) (E1A1002 and E1A3089, EnoGene, China); rabbit monoclonal anti-phospho-Stat3 (Tyr705) (#9145, Cell Signaling Technology, Beverly, MA, USA). Monoclonal mouse IgG2a and ployclonal rabbit IgG (X0943 and X0936, DAKO A/S, Glostrup, Denmark) were used as isotope controls.

A total of 10 to 20 areas (depending on the size of the section) at 400× magnification of each section were examined under microscopy, and the immunostaining was assessed by two researchers independently in a blinded fashion, i.e., without the knowledge of clinic pathologic information. The expression level of ISL-1 in lymphomas was scored semi-quantitatively based on the percentage of positive cells and classified into negative (<25% neoplastic cells stained positive), weak (25 ~ 49% neoplastic cells stained positive), moderate (50 ~ 75% neoplastic cells stained positive) and strong staining (>75% neoplastic cells stained positive), which represent the score of 0, 1, 2 and 3. The images were acquired with a Leica DM25000B microscope (Leica, Germany). The association between immunoreactivity and patient clinic pathological parameters was assessed by χ^2^ test. Positive percent was determined by number of strong staining samples (with ISL-1 expression level scored 2 or 3) over the total number of samples.

### Cell culture and the establishment of stable cell lines

Diffuse large B cell lymphoma cell lines SUDHL16 and Ly3 (gifts from Prof. Zhu J, Peking University Cancer Hospital), Burkitt lymphoma cell lines (Raji, preserved by our laboratory, and Ramos purchased from Cell Resource Center of Shanghai Institutes for Biological Science, Shanghai, China), T-cell leukemia/lymphoma cell line Jurkat (kindly provided by Prof. Ma DL, Peking University School of Basic Medical Sciences) were grown in RPMI 1640 containing 10% fetal bovine serum. HeLa cells (preserved by our laboratory) were cultured in Dulbecco’s modified eagle medium supplemented with 10% fetal calf serum.

To establish stable cell lines with ISL-1 overexpressing, Raji, Ly3 and Jurkat cells were transfected with 10 μg pcDNA3.1-ISL1 plasmid [6], or a control pcDNA3.1 plasmid, at a density of 1 × 10^7^/ml, in a 0.2 cm cuvette (BTX) using an BTX ECM803 Electroporator (Genetronics, San Diego, CA, USA) at 130 V, 20 ms. G418 selection (1000 μg/ml) was performed 48 h after transfection. The cells were cultured for about 21 days to obtain stable cell lines. The culture medium containing G418 was changed every two days. To establish stable knockdown cell lines, Ly3 and Jurkat cells were electroporated with pLL3.7-ISL1-siRNA or pLL3.7-nonsilencer, respectively [6], and 48 h after transfection, cells were cultured with a puromycin-containing medium (0.5 μg/ml) for about 21 days.

### Cell treatment, cell proliferation and cell cycle assays

STATTIC (sc-202818, Santa Cruz Biotechnology, Santa Cruz, CA, USA), SP600125 (s5567) and Anisomycin (A9789) purchased from Sigma (Louis, MO, USA) were dissolved in 100% dimethyl sulfoxide to prepare a 40 mM stock, 20 mM stock and 15 mg/ml stock respectively, and stored at -20°C. Recombinant human interleukin (IL)-6 purchased from R & D Systems (206IL, Minneapolis, MN, USA) was reconstituted in sterile PBS containing 0.1% bovine serum albumen to prepare a 10 μg/ml stock and stored at -20°C. The stock solution was added in culture medium to achieve the indicated final concentrations for cell culture.

The cell proliferation was examined using a CCK-8 cell proliferation kit (Dojindo Laboratories, Kumamoto, Japan), according to the instruction from the supplier. Absorbance was measured at 450 nm with Microplate Reader (Bio-Rad, La Jolla, CA, USA). After 24 hr serum depletion (0.2% FBS), cells were subsequently incubated in 10% FBS medium for an additional 24 hr before harvest. Cell cycle assessment and data analysis were carried out referring to our previous report [6] using FACS Calibur flow cytometry (Becton Dickinson, Franklin Lakes, NJ, USA) equipped with the ModiFit LT v2.0 software.

### Animal experiments

The animal experiments were performed in accordance with the ethical principles and guidelines for scientific experiments on animals of the Swiss Academy of Medical Sciences (1995). All protocols were approved by the Animal Care and Use Committee of Peking University (LA 2010–066). Five-week-old NOD/SCID (nonobese diabetic/severe combined immune deficient) mice were purchase from the Department of Laboratory Animal Science of Peking University. Six or five mice per group were injected subcutaneously into the left and right oxter flank with 1 × 10^7^ cells resuspended in 200 μl PBS. All mice were maintained under specific pathogen-free conditions. Tumors were measured at indicated time with calipers and tumor volumes were calculated as 1/2 × length × width^2^. The mice were sacrificed by euthanasia just before tumor skin festering and the tumors were collected for further analysis. The significance of differences between groups was determined using the 2-way ANOVA.

### Luciferase assays

The plasmid transfection and luciferase activity detection were performed as described before [30]. *ISL-1-luc* plasmid was constructed previously by our laboratory [30]. pCDNA3.1-c-Myc, *c-Myc-luc* was kindly provided by Prof. Shang YF, Peking University School of Basic Medical Sciences. *c-Myc-luc* M1, M2, D1 and D2 were commercially constructed by TransGen Biotech (Beijing, China).

### RT-PCR and real-time RT-PCR

Total RNA was extracted using Trizol Reagent (Invitrogen, Carlsbad, CA, USA) based on the manufacturer’s instructions. RT-PCR and real-time RT-PCR amplifications were performed as descripted before [30] with different primers: ISL-1: F: 5′-CTGCTTTTCAGCAACTGGTCA-3′, R: 5′-TAGGACTGGCTACCATGCTGT-3′; c-Myc: F: 5′-GCCACGTCTCCACACATCAG-3′, R: 5′-TCTTGGCAGCAGGATAGTCCTT-3′; GAPDH: F: 5′-CGACCACTTTGTCAAGCTCA-3′, R: 5′-AGGGGTCTACATGGCAACTG-3′.

### Immunoprecipitation and Western blot analysis

Cell lysates were prepared using RIPA lysis buffer (P0013E, Beyotime, Shanghai, China), which included protease and phosphatase inhibitors (469313200, 14906845001, Roche, Basle, Switzerland). Immunoprecipitation and Western blot analysis were carried out as described before using the indicated antibody [49]. Mouse monoclonal anti-ISL-1 (H00003670-M05, Abnova, Taipei, China); rabbit monoclonal anti-ISL-1 (3727–1, Eptomics an abcam company, Hong Kong, China); rabbit monoclonal anti-phosphor-JNK (Thr163/Tyr165) (#4668), rabbit monoclonal anti-c-Myc (#5605), rabbit monoclonal anti-STAT3 and anti-phospho-STAT3 (Tyr705) (#4904 and #9145), rabbit monoclonal anti-GAPDH (#2118) and anti-β-tubulin (#2146) were all purchased from Cell Signaling Technology (Beverly, MA, USA). Mouse monoclonal anti-phospho-c-Jun (Ser63) (sc-822) and rabbit polyclonal anti-c-Myc (sc-764) were obtained from Santa Cruz Biotechnology (Santa Cruz, CA, USA).

### Chromatin immunoprecipitation (ChIP) and ChIP-re-IP assays

ChIP and ChIP-re-IP experiments were performed in Ly3 cells according to the method described by Zhang Y *et al.*[50] using primers covering ISL-1 promoter region: Primer 1: F: 5′-CCTTTCCTCCCACCCAACGTTTTTA-3′, R: 5′-GCTTGGTTTGGTCCCCACG-3′; Primer 2: F: 5′-GTGGGGACCAAACCAAGCTGAAC-3′, R: 5′-GGTCCCCGCAGTCCGGCT-3′; Primer 3: F: 5′-AGGAGCAGCGCCACAGGAG-3′, R: 5′-ATTATCATATTTCAGCCTCGCCGC-3′; Primer 4: F: 5′-GCGGCGAGGCTGAAATATGATAAT-3′, R: 5′-GCAGACTCGGCGCGGCTC-3′; or primer covering c-Myc enhancer region: c-Myc: F: 5′-TACAGTGCACTTTCACTAGTATTCA-3′, R: 5′-TTATTGGAAATGCGGTCATG-3′.

### Statistical analysis

Statistical analyses were carried out using the statistical software SPSS 17.0. The data are expressed as means ± standard deviation (S.D.). Differences were considered to be statistically significant at *p* < 0.05.

## Competing interests

The authors declare no conflict of interests.

## Authors’ contributions

QZ, WW and CZ conceived and designed the experiments; QZ, ZY and CG performed the experiments and analyzed the data; PC, KM, ZJ, HL and CL contributed reagents/materials/analysis tools; QZ, ZY, WW and CZ wrote the paper. All authors read and approved the final manuscript.

## Supplementary Material

Additional file 1: Figure S1 ISL-1 is efficiently overexpressed or knockdown in stably transfected NHL cells. ISL-1 expression was detected by Western blot analysis in stably transfected Raji **(A)**, Ly3 **(B)** and Jurkat **(C)** cells. GAPDH served as an internal control. Representative images are shown (**A** to **C** top panel) and the degree of ISL-1 expression changes was calculated by gray scanning (**A** to **C** bottom panel) using the Bio-Rad Quantity One software on the Western images from 3 independent experiments. Each bar represents mean ± SD. *p* values were calculated using a Student *t*-test (*p<0.05, **p<0.01, ##*p*<0.01, ###*p*<0.001). Click here for file

Additional file 2: Figure S2 The ISL-1-overexpressing or -knockdown cells produce significantly larger or smaller tumors than the control or non-silencer cells. NOD-SCID mice were injected s.c. with different NHL cells that were stably transfected with pcDNA3.1 (Control), or pcDNA3.1-ISL-1 (ISL-1) construct **(A,C)**, pLL3.7-Non-silencer or pLL3.7-ISL1-siRNA plasmid **(B,D)**. The mice were killed after the last measurement of tumor volume and the tumors were isolated and weighed. Statistical analysis was carried out with 2-way ANOVA (*p<0.05, **p<0.01). Click here for file

Additional file 3: Figure S3 ISL-1 expression level can be specifically regulated by JNK and JAK/STAT signaling pathways. Western blot showed the expression changes of ISL-1 in NHL cells after treated with WNT signaling pathway inhibitor or activator (Frizzle, 10 μM or WNT3a protein, 100 ng/ml), MAPK/ERK signaling pathway inhibitor (PD98059, 10 μM), P38/MAPK signaling pathway inhibitor (SB203580, 10 μM), SAPK/JNK signaling pathway inhibitor (SP600125, 10 μM), or JAK/STAT signaling pathway inhibitor (STATTIC, 6 μM) for 24 h. The degree of ISL-1 expression changes was calculated by gray scanning using the Bio-Rad Quantity One software on the Western images. The data represent three independent experiments, each performed in triplicate. Each bar represents mean ± SD (*p<0.05). Click here for file

Additional file 4: Figure S4 The phosphorylation of c-Jun or STAT3 can be inhibited by the specific JNK or JAK/STAT signaling pathway inhibitor, respectively. Ly3 cells were treated with JNK signaling pathway inhibitor (SP60012, 10 μM) or JAK/STAT signaling pathway inhibitor (STATTIC, 6 μM) for indicated time. The inhibitory effect of SP600125 on c-Jun phosphorylation **(A)** and the inhibitory effect of STATTIC on p-STAT3 phosphorylation **(B)** were analyzed by Western blot. GAPDH served as an internal control. Click here for file
