# Supplementary material for: ISL-1 is overexpressed in non-Hodgkin lymphoma and promotes lymphoma cell proliferation by forming a p-STAT3/p-c-Jun/ISL-1 complex
Source: Mol Cancer. 2014 Jul 29;13:181. doi: 10.1186/1476-4598-13-181 (PMC4125377; doi:10.1186/1476-4598-13-181)
Supplement: Additional file 1: Figure S1 — ISL-1 is efficiently overexpressed or knockdown in stably transfected NHL cells. ISL-1 expression was detected by Western blot analysis in stably transfected Raji (A), Ly3 (B) and Jurkat (C) cells. GAPDH served as an internal control. Representative images are shown (A to C top panel) and the degree of ISL-1 expression changes was calculated by gray scanning (A to C bottom panel) using the Bio-Rad Quantity One software on the Western images from 3 independent experiments. Each bar represents mean ± SD. p values were calculated using a Student t-test (*p<0.05, **p<0.01, ##p<0.01, ###p<0.001). [file 1476-4598-13-181-S1.doc]

**Addtional file 1: Figure S1**


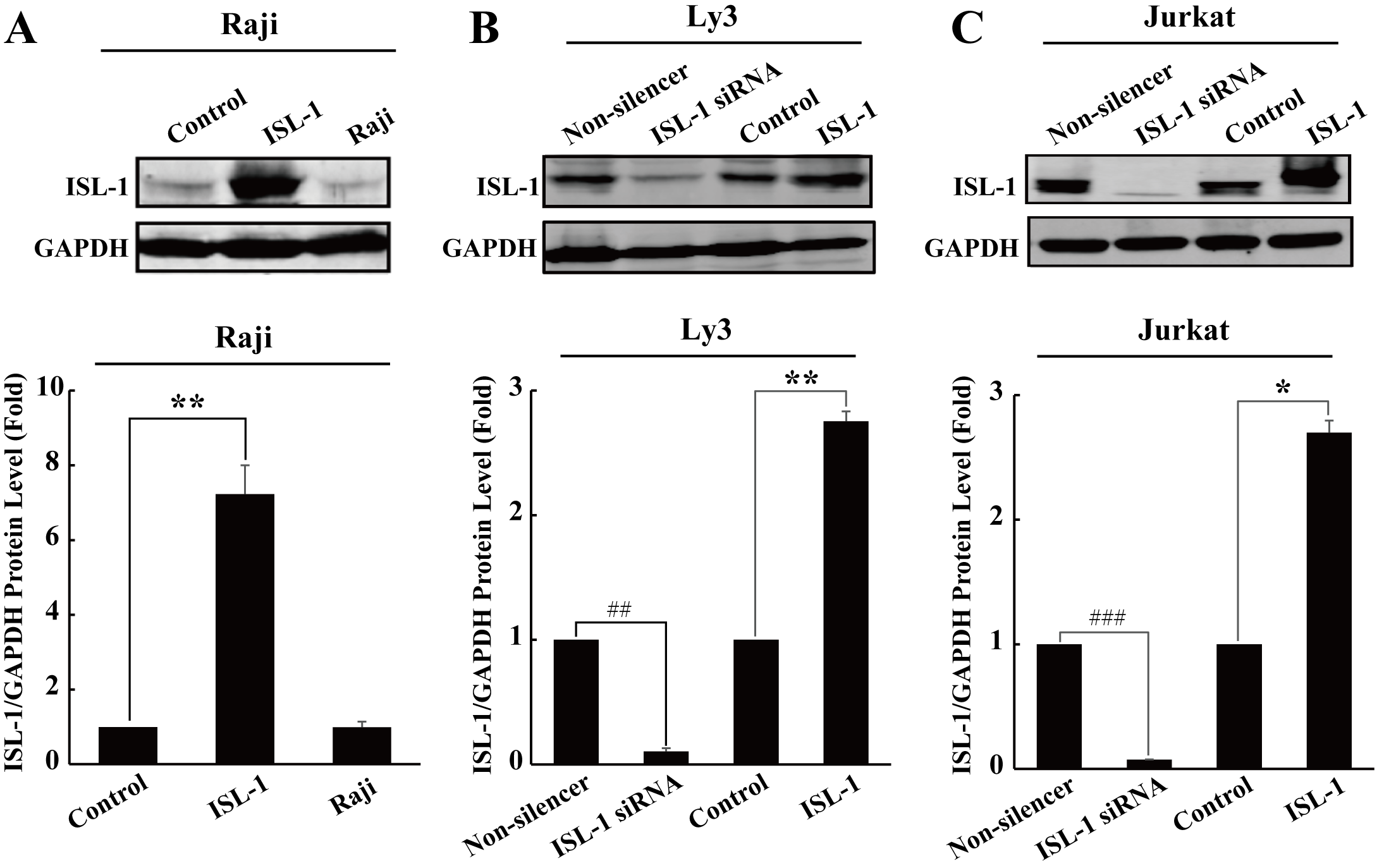


**Figure S1 ISL-1 is efficiently overexpressed or knockdown in stably transfected NHL cells.** ISL-1 expression was detected by Western blot analysis in stably transfected Raji (**A**), Ly3 (**B**) and Jurkat (**C**) cells. GAPDH served as an internal control. Representative images are shown (**A to C top panel**) and the degree of ISL-1 expression changes was calculated by gray scanning (**A to C bottom panel**) using the Bio-Rad Quantity One software on the Western images from 3 independent experiments. Each bar represents mean ± SD. *p* values were calculated using a Student *t*-test (**p*<0.05, ***p*<0.01, ##*p*<0.01, ###*p*<0.001).
