# Supplementary material for: ISL-1 is overexpressed in non-Hodgkin lymphoma and promotes lymphoma cell proliferation by forming a p-STAT3/p-c-Jun/ISL-1 complex
Source: Mol Cancer. 2014 Jul 29;13:181. doi: 10.1186/1476-4598-13-181 (PMC4125377; doi:10.1186/1476-4598-13-181)
Supplement: Additional file 2: Figure S2 — The ISL-1-overexpressing or -knockdown cells produce significantly larger or smaller tumors than the control or non-silencer cells. NOD-SCID mice were injected s.c. with different NHL cells that were stably transfected with pcDNA3.1 (Control), or pcDNA3.1-ISL-1 (ISL-1) construct (A,C), pLL3.7-Non-silencer or pLL3.7-ISL1-siRNA plasmid (B,D). The mice were killed after the last measurement of tumor volume and the tumors were isolated and weighed. Statistical analysis was carried out with 2-way ANOVA (*p<0.05, **p<0.01). [file 1476-4598-13-181-S2.doc]

**Additional file 2: Figure S2**

**
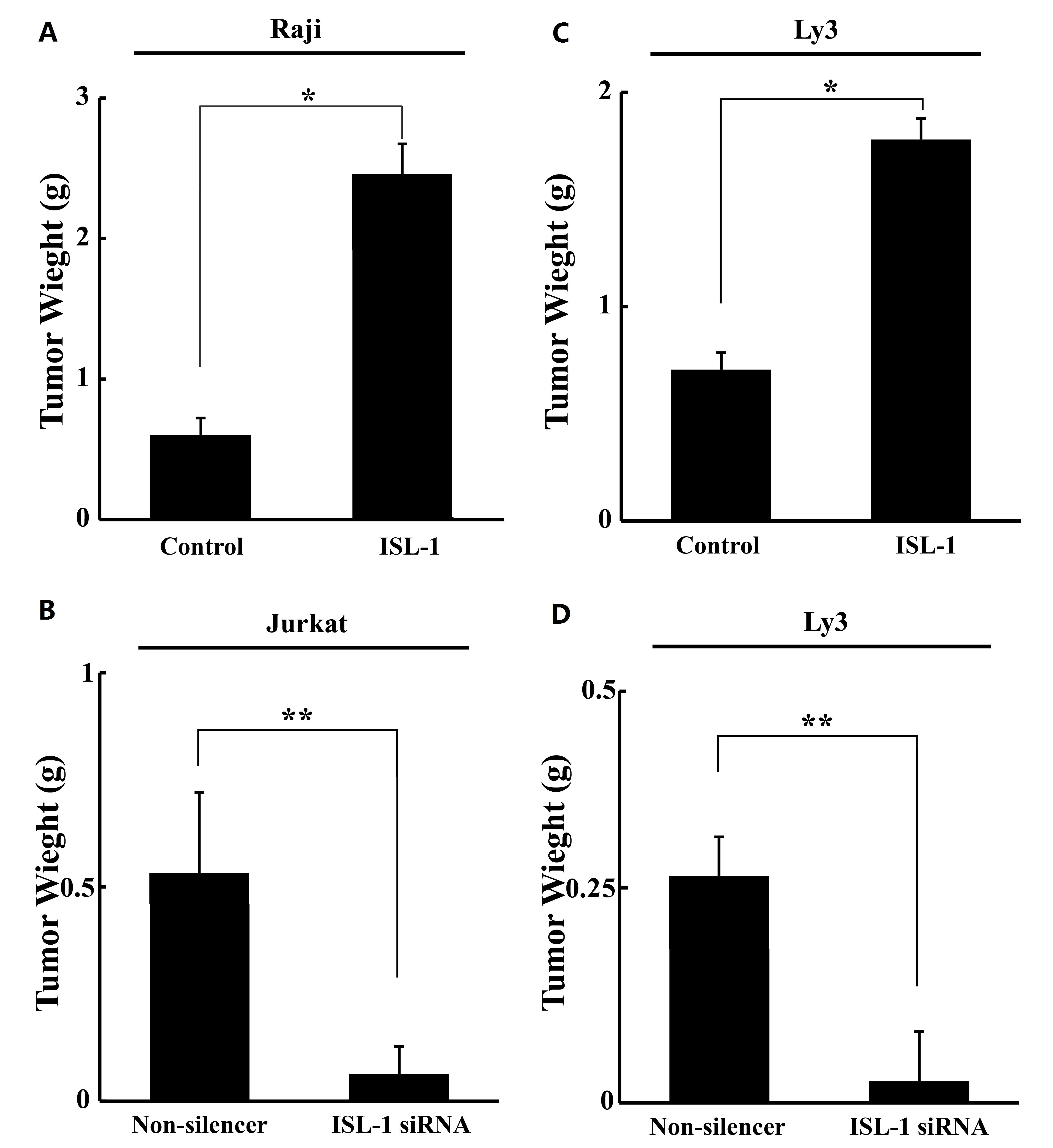
**

**Figure S2**  **The ISL-1-overexpressing or -knockdown cells produce significantly larger or smaller tumors than the control or non-silencer cells.** NOD-SCID mice were injected s.c. with different NHL cells that were stably transfected with pcDNA3.1 (Control), or pcDNA3.1-ISL-1 (ISL-1) construct (**A, C**), pLL3.7-Non-silencer or pLL3.7-ISL1-siRNA plasmid (**B, D**). The mice were killed after the last measurement of tumor volume and the tumors were isolated and weighed. Statistical analysis was carried out with 2-way ANOVA (**p*<0.05, ***p*<0.01).
