# Supplementary material for: ISL-1 is overexpressed in non-Hodgkin lymphoma and promotes lymphoma cell proliferation by forming a p-STAT3/p-c-Jun/ISL-1 complex
Source: Mol Cancer. 2014 Jul 29;13:181. doi: 10.1186/1476-4598-13-181 (PMC4125377; doi:10.1186/1476-4598-13-181)
Supplement: Additional file 3: Figure S3 — ISL-1 expression level can be specifically regulated by JNK and JAK/STAT signaling pathways. Western blot showed the expression changes of ISL-1 in NHL cells after treated with WNT signaling pathway inhibitor or activator (Frizzle, 10 μM or WNT3a protein, 100 ng/ml), MAPK/ERK signaling pathway inhibitor (PD98059, 10 μM), P38/MAPK signaling pathway inhibitor (SB203580, 10 μM), SAPK/JNK signaling pathway inhibitor (SP600125, 10 μM), or JAK/STAT signaling pathway inhibitor (STATTIC, 6 μM) for 24 h. The degree of ISL-1 expression changes was calculated by gray scanning using the Bio-Rad Quantity One software on the Western images. The data represent three independent experiments, each performed in triplicate. Each bar represents mean ± SD (*p<0.05). [file 1476-4598-13-181-S3.doc]

**Additional file 3: Figure S3**

**
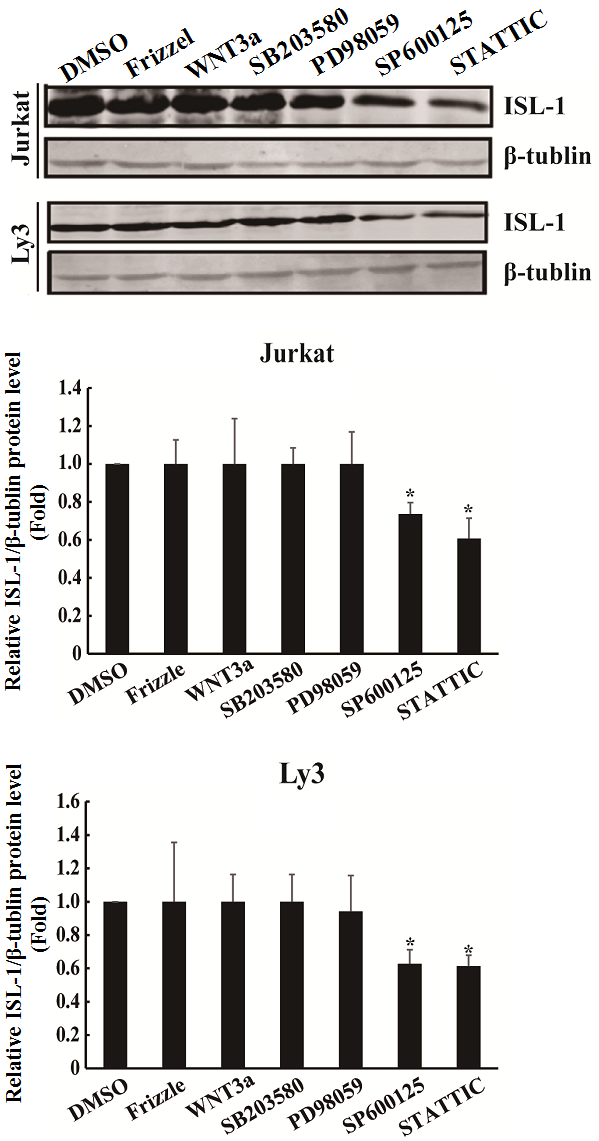
**

**Figure S3 ISL-1 expression level can be specifically regulated by JNK and JAK/STAT signaling pathways.** Western blot showed the expression changes of ISL-1 in NHL cells after treated with WNT signaling pathway inhibitor or activator (Frizzle, 10 μM or WNT3a protein, 100 ng/ml), MAPK/ERK signaling pathway inhibitor (PD98059, 10 μM), P38/MAPK signaling pathway inhibitor (SB203580, 10 μM), SAPK/JNK signaling pathway inhibitor (SP600125, 10 μM), or JAK/STAT signaling pathway inhibitor (STATTIC, 6 μM) for 24 h. The degree of ISL-1 expression changes was calculated by gray scanning using the Bio-Rad Quantity One software on the Western images. The data represent three independent experiments, each performed in triplicate. Each bar represents mean ± SD (*p<0.05).
