# Supplementary material for: ISL-1 is overexpressed in non-Hodgkin lymphoma and promotes lymphoma cell proliferation by forming a p-STAT3/p-c-Jun/ISL-1 complex
Source: Mol Cancer. 2014 Jul 29;13:181. doi: 10.1186/1476-4598-13-181 (PMC4125377; doi:10.1186/1476-4598-13-181)
Supplement: Additional file 4: Figure S4 — The phosphorylation of c-Jun or STAT3 can be inhibited by the specific JNK or JAK/STAT signaling pathway inhibitor, respectively. Ly3 cells were treated with JNK signaling pathway inhibitor (SP60012, 10 μM) or JAK/STAT signaling pathway inhibitor (STATTIC, 6 μM) for indicated time. The inhibitory effect of SP600125 on c-Jun phosphorylation (A) and the inhibitory effect of STATTIC on p-STAT3 phosphorylation (B) were analyzed by Western blot. GAPDH served as an internal control. [file 1476-4598-13-181-S4.doc]

**Additional file 4: Figure S4**

**
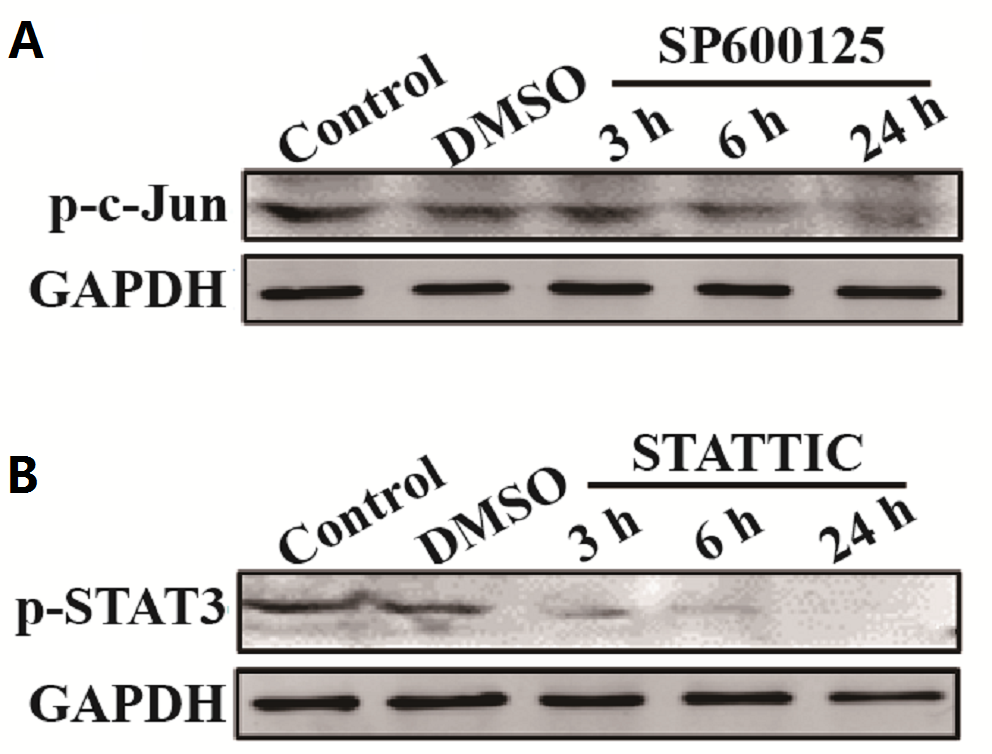
**

**Figure S4 The phosphorylation of c-Jun or STAT3 can be inhibited by the specific JNK or JAK/STAT signaling pathway inhibitor, respectively.** Ly3 cells were treated with JNK signaling pathway inhibitor (SP60012, 10 μM) or JAK/STAT signaling pathway inhibitor (STATTIC, 6 μM) for indicated time. The inhibitory effect of SP600125 on c-Jun phosphorylation (A) and the inhibitory effect of STATTIC on p-STAT3 phosphorylation (B) were analyzed by Western blot. GAPDH served as an internal control.
